# Racial differences in cardiopulmonary outcomes of hospitalized COVID-19 patients with acute kidney injury

**DOI:** 10.31083/j.rcm2204174

**Published:** 2021-12-22

**Authors:** Obiora Egbuche, Temidayo Abe, Shirley I. Nwokike, Opeyemi Jegede, Kenechukwu Mezue, Titilope Olanipekun, Ifeoma Onuorah, Melvin R. Echols

**Affiliations:** 1 Division of Cardiovascular Medicine, Ohio State University, Columbus, OH 43210, USA; 2 Department of Internal Medicine, Morehouse School of Medicine, Atlanta, GA 30310, USA; 3 Department of Internal Medicine, Medical College of Georgia, Augusta, GA 30912, USA; 4 Department of Biostatistics and Epidemiology, University of North Texas Health Science Center, Fort-Worth, TX 76107, USA; 5 Division of Nuclear Cardiology, Massachusetts General Hospital, Harvard Medical School, Boston, MA 02114, USA; 6 Department of Hospital Medicine, Covenant Health System, Knoxville, TN 37902, USA; 7 Division of Cardiovascular Disease, Emory University School of Medicine, Atlanta, GA 30322, USA; 8 Division of Cardiovascular Medicine, Morehouse School of Medicine, Atlanta, GA 30310, USA

**Keywords:** Black race, Cardiac events, AKI, Mortality, COVID-19

## Abstract

In-hospital acute kidney injury (IH-AKI) has been reported in a significant proportion of patients with COVID-19 and is associated with increased disease burden and poor outcomes. However, the mechanisms of injury are not fully understood. We sought to determine the significance of race on cardiopulmonary outcomes and in-hospital mortality of hospitalized COVID-19 patients with AKI. We conducted a retrospective cohort study of consecutive patients hospitalized in Grady Health System in Atlanta, Georgia between February and July 2020, who tested positive for severe acute respiratory syndrome coronavirus-2 (SARS-CoV-2) on qualitative polymerase-chain-reaction assay. We evaluated the primary composite outcome of in-hospital cardiac events, and mortality in blacks with AKI versus non-blacks with AKI. In a subgroup analysis, we evaluated the impact of AKI in all blacks and in all non-blacks. Of 293 patients, effective sample size was 267 after all exclusion criteria were applied. The mean age was 61.4 ± 16.7, 39% were female, and 75 (28.1%) had IH-AKI. In multivariable analyses, blacks with IH-AKI were not more likely to have in-hospital cardiac events (aOR 0.3, 95% Confidence interval (CI) 0.04–1.86, *p*=0.18), require ICU stay (aOR 0.80, 95% CI 0.20–3.25, *p* = 0.75), acute respiratory distress syndrome (aOR 0.77, 95% CI 0.16–3.65, *p* = 0.74), require mechanical ventilation (aOR 0.51, 95% CI 0.12–2.10, *p* = 0.35), and in-hospital mortality (aOR 1.40, 95% CI 0.26–7.50, *p* = 0.70) when compared to non-blacks with IH-AKI. Regardless of race, the presence of AKI was associated with worse outcomes. Black race is not associated with higher risk of in-hospital cardiac events and mortality in hospitalized COVID-19 patients who develop AKI. However, blacks with IH-AKI are more likely to have ARDS or die from any cause when compared to blacks without IH-AKI.

## 1. Introduction

Acute kidney injury (AKI) frequently occurs among patients with COVID-19 disease [1]. It may occur early, in temporal association with respiratory failure, and is associated with poor prognosis [2]. The frequent development of IH-AKI with COVID-19 has brought into focus the pathophysiologic mechanisms, racial disparities, and clinical outcomes in COVID-19 patients. The reported incidence of IH-AKI in patients with COVID-19 is approximately 3%–29%, with wide variations between cohorts observed [3–7]. A report noted that this range may be from 0.5% to 80% [1]. The wide range of reported incidence is likely due to inconsistencies in AKI definitions [8], variability in incidence of people hospitalized with mild symptoms versus critically ill patients [1, 8], and difference in geographic locations [1]. Variability in patient demographics such as race/ethnicity has also been cited as an explanation for this wide variation in AKI incidence [1]. The pathogenesis of IH-AKI in COVID-19 is unclear due to limited availability of kidney biopsy reports [8]. However, affectation of different kidney compartments including the glomeruli, tubules and vascular components have been proposed as the explanation for the various clinical presentations that include syndrome of inappropriate antidiuretic hormone secretion, collapsing glomerulopathy, cardiorenal syndrome, thrombotic microangiopathy, and acute kidney disease [1]. There is limited literature that explain the differential affectation of the different functional compartments of the kidney in specific individuals or race.

The COVID-19 pandemic remains an ongoing cause of global morbidity and mortality [9]. SARS-CoV-2, the etiologic agent of COVID-19, is an enveloped single-stranded RNA virus which may cause mild or no symptoms at all. However, respiratory illness such as severe acute respiratory distress syndrome (ARDS), and in the most severe cases, fatal multi-organ failure including AKI, and death may occur [10]. As of May 12, 2021, there were 159,319,384 confirmed cases and 3,311,780 reported deaths worldwide [11]. According to the World Health Organization, there have been a total of 32,424,637 confirmed cases and 576,814 reported deaths in the United States as of May 12, 2021 [11]. As prevalent cases of COVID-19 increase, the burden of AKI and its attendant complications increase. Available literature report that about 3% to 17% of COVID-19 patients who develop AKI will require renal replacement therapy [6, 7]. This increases morbidity and mortality, as well as healthcare cost. Therefore, emphasis should be laid on the risk factors for development of AKI, its pathophysiologic mechanisms, racial predilection, optimal therapy, and prevention in the COVID-19 patient population.

Several studies suggest significantly higher transmission and hospitalization rates in blacks when compared with other populations [12–14]. Although some studies have suggested an association of black race with worse outcomes in COVID-19 patients [13, 14], and black race has been reported as a risk factor for development of AKI in COVID-19 patients [2], there is limited data as to why this association is seen.

To date, the available literature on the racial differences in clinical outcomes of IH-AKI in COVID-19 patients is limited. Overall, the rates of IH-AKI in COVID-19 patients and associated outcomes are not fully understood [2]. Black patients with COVID-19 generally have worse outcomes [13, 14] and the presence of IH-AKI in COVID-19 portends a poorer prognosis [8]. Black race, among others, have also been cited as a risk factor for development of AKI in patients with COVID-19 [2]. Thus, the objective of this study is to evaluate the impact of race on cardiopulmonary outcomes and mortality in hospitalized COVID-19 patients with AKI.

## 2. Methods

This retrospective cohort study included patients seen at Grady Health System between February 1 and July 31, 2020, who tested positive for SARS-CoV-2 on qualitative polymerase chain-reaction assay and were hospitalized for symptomatic COVID-19 infection. This cohort included all hospitalized patients, regardless of race, admitted for acute COVID-19 infection. The study cohort was stratified by race into 2 sub-groups, blacks and non-blacks. The race/ethnicity of the patients was obtained from the electronic medical record of the health system as previously reported by the patients themselves. An ethical approval from Morehouse School of Medicine institutional review board (IRB) and Grady Health System ethical committee was obtained.

### 2.1 Data collection

Clinical data were extracted by trained graduate and undergraduate medical education physicians from the health system’s electronic medical record system, Epic. The data extracted included the following: demographic characteristics (patient-reported race, age, sex, ethnic group, insurance status), body-mass index, medication use (statin use, calcium channel blockers, aspirin, beta-blockers, P2Y12 platelet inhibitors, angiotensin-converting enzyme [ACE] inhibitors, angiotensin receptor blockers [ARBs]), chronic medical conditions, data from serological testing, in-hospital therapies including any use of non-invasive or mechanical ventilation, and clinical symptoms during admission. Three blinded independent physician investigators ensured quality control of the extracted data. These investigators randomly selected patients from the study cohort and adjudicated the extracted datapoints. COVID-19 was confirmed with a viral nucleic acid [reverse-transcriptase-polymerase-chain-reaction (RTPCR)] assay of samples obtained from nasopharyngeal swab. Patients were included in the analysis only if they were admitted for symptom management due to a positive test result. The study did not include patients if they tested positive for the virus were stable enough to be managed on outpatient basis. AKI was defined according to the Kidney Disease Improving Global Outcomes (KDIGO) guideline as increase in serum creatinine by 0.3 mg/dL or more within 48 hours; or increase in serum creatinine up to or more than 1.5 times baseline which is known or presumed to have occurred within the prior 7 days [15]. There was no sub-stratification into the various stages of AKI. Individuals with pre-existing end stage renal disease (ESRD) were intended to be excluded from the analyses, however no ESRD patients were identified or included in our study cohort. Acute respiratory distress syndrome (ARDS) was defined according to the 2013 Berlin definition [16]. Myocardial injury was defined as blood levels of cardiac biomarker (troponin) above the 99th-percentile upper reference limit. Myocardial infarction was defined as myocardial injury in the presence of either typical angina, new ischemic ST-T wave changes on electrocardiography, new regional wall motion abnormality on echocardiography, or angiographic evidence of an acute coronary event. Study-specific outcome measures were monitored up to August 25, 2020 or the last day of follow-up.

### 2.2 Outcome measures and covariates

The primary outcome measure was a composite of acute in-hospital cardiac events which included any of acute heart failure, cardiogenic shock, fatal and non-fatal arrhythmias, myocardial injury, acute myocardial infarction, or death from any cause. The secondary outcomes measures included myocardial injury, in-hospital mortality, ICU stay, and ARDS. In a subgroup analysis, we evaluated the impact of AKI on outcomes in all-comers, blacks only, and non-blacks only.

### 2.3 Statistical analysis

Baseline characteristics of the patients who developed in-hospital AKI versus the whole study cohort were evaluated. Descriptive analyses were done using appropriate measures of location, dispersion, and proportion. Patients who tested positive for COVID-19 virus but not hospitalized were excluded from the study. Patients who identified themselves as Hispanic, Asian, white, or other ethnic group were classified as non-blacks. Independent sample *T*-test (or Wilcoxon Rank Sum test where appropriate) and Chi-square (or Fisher’s exact where appropriate) tests were used to compare continuous and binary variables respectively by the levels of the primary and secondary outcome variables. All outcome variables were defined on a binary scale. In univariable analysis, Breslow-Day test was used to assess the homogeneity of odds ratios for the association between AKI and outcomes by levels of race (black vs non-blacks). In multivariable analysis, the odds ratios for the association between race and outcomes in patients with AKI were evaluated. A race by AKI interaction term was included and assessed (for statistical significance) in a binary logistic regression. Covariates adjusted for in the multivariable logistic regression include demographic characteristics including gender and age, as well as comorbidities including pCVD, diabetes mellitus, hypertension, chronic obstructive pulmonary disease (COPD), peripheral artery disease, chronic kidney disease (CKD), dyslipidemia, obesity, asthma. No multiple imputation was made for missing data. All data analysis was performed using SAS v9.4 software (SAS Institute Inc., Cary, NC, USA). For all the statistical analyses, 2-sided *p*-value ≤ 0.05 was considered significant.

## 3. Results

### 3.1 Demographics

Data was collected on 293 hospitalized patients. Of these 293 patients, 21 were excluded for missing datapoints. Of the remaining 272, 222 were blacks and 50 were non-blacks. For analysis including all-comers, another 5 patients were excluded for missing specific covariates. Of these 267 patients, 232 patients were successfully treated and discharged, and 35 (13.1%) patients died. This cohort included 163 (61%) men and 104 (39%) women, with a mean ± SD age of all included patients 61.4 ± 16.7. Of the 267 patients, 75 (28.1%) developed AKI during hospitalization. The rate of AKI in the black cohort was 66/222 (30%) and was 9/50 (18%) in the non-black cohort. Baseline characteristics with descriptive analyses are summarized in Table 1.

### 3.2 Treatment variables

In-hospital treatment variables are listed in Table 2. Of the 267 patients, 154 (58%) required oxygen support, 91 (34%) required non-invasive ventilation, and 50 (19%) of the cohort required mechanical ventilation during the hospitalization. There was a higher proportion of patients in the AKI group who required oxygen support, non-invasive ventilation, or mechanical ventilation when compared to proportions in the whole study cohort. In terms of medical treatment, 43 (16%) patients received Hydroxychloroquine, while 54 (20%) patients received Remdesivir. Corticosteroids were administered to 46 (17%) of all patients.

### 3.3 In-hospital cardiopulmonary endpoints

The proportions of study outcomes of IH-AKI by race are listed in Table 3. Overall, there was no statistically significant difference in the cardiopulmonary outcomes on in-hospital AKI between blacks and non-blacks. Black patients who develop AKI during hospitalization for COVID-19 are equally likely to have the primary composite outcome of cardiac events (aOR 0.3, 95% CI 0.04–1.86, *p* = 0.18), in-hospital death (aOR 1.40, 95% CI 0.26–7.50, *p* = 0.70), ARDS (aOR 0.77, 95% CI 0.16–3.65, *p* = 0.74), and require ICU stay (aOR 0.80, 95% CI 0.20–3.25, *p* = 0.75) when compared to non-blacks (Table 3).

In adjusted analyses, IH-AKI was associated with all cardiopulmonary outcomes in analysis including all study participants and in subgroup analysis of blacks (Table 4). In specific cohorts, IH-AKI was statistically associated with the primary composite outcome in all study participants (aOR 2.9, 95% CI 1.6–5.3, *p* = 0.0004), blacks (aOR 2.7, 95% CI 1.3–5.5, *p* = 0.006), and non-blacks (aOR 8.6, 95% CI 1.3–54.8, *p* = 0.02) (Table 4). In independent adjusted subgroup analyses, IH-AKI was statistically associated with in-hospital death in blacks (aOR 8.9, 95% CI 3.3–23.9, *p <* 0.0001), but not in non-blacks (aOR 11.4, 95% CI 0.9–143.6, *p* = 0.06) (Table 4). There was no statistically significant race by AKI interaction term.

Black patients with in-hospital AKI are more likely to have ARDS (aOR 6.4, 95% CI 3.1–13.1, *p <* 0.0001) when compared to blacks without AKI. On the contrary, non-blacks with IH-AKI did not have increased odds of developing ARDS (aOR 4.3, 95% CI 0.9–20.3, *p* = 0.07) when compared to non-blacks without AKI. When compared to black patients without AKI, black patients with IH-AKI are more likely to have myocardial injury (aOR 2.2, 95% CI 1.1–4.3, *p* = 0.025) when hospitalized for COVID-19. However, in non-blacks, IH-AKI is not associated with myocardial injury (aOR 4.3, 95% CI 0.8–22.6, *p* = 0.08) (see Table 4). Of the 75 patients who developed AKI, 14 (18.7%) required renal replacement therapy (Table 5). Of these 14 patients, 11 (78.6%) were blacks. The mortality rate among blacks with AKI requiring renal replacement therapy is 7/11 (63.6%) compared to 1/3 (33.3%) among non-blacks (Table 6). The case fatality rate (CFR) of COVID-19 in our study was 35/267 (13.1%). However, among patients who developed IH-AKI, the CFR increases to 22/75 (29.3%). In black patients who develop IH-AKI during an admission for COVID-19, the CFR is 20/66 (30%) compared to 2/9 (22%) in non-blacks. However, there was no statistical racial difference in mortality outcomes (aOR 1.40, 95% CI 0.26–7.50, *p* = 0.70) (Table 3). Overall, IH-AKI portend worse cardiopulmonary hard outcomes in COVID-19 infection. However, in patients who develop IH-AKI, there were no observed racial differences in outcomes.

## 4. Discussion

In this retrospective study conducted in a tertiary safety-net hospital serving predominantly black patients as well as patients from lower socioeconomic strata, we noted that 28.1% of hospitalized patients with COVID-19 developed IH-AKI prior to discharge. Our reported incidence of IH-AKI among COVID-19 patients is similar and within the range on incidence reported in studies conducted in other racial populations [3–7, 17]. A study of predominantly underserved African Americans reported AKI rates as high as 49.3% [18]. In blacks and non-blacks, our study suggests that patients who developed IH-AKI during the admission were more likely to experience a cardiac event, require ICU stay, and mechanical ventilation compared to patients with stable renal function. This is also true when all study participants are considered as a group. Blacks who develop in-hospital AKI have higher odds of having ARDS, myocardial injury, or die from any cause. Unlike in non-blacks where AKI does not portend higher odds for these 3 outcomes. Albeit, the event rate in the non-black cohort may have been under estimated due to smaller sample size. However, in patients who develop AKI, black race does not add any incremental risk of adverse outcomes.

Although ARDS and respiratory failure were considered the main causes of admission to an intensive care unit in patients with COVID-19, IH-AKI independently contributes significantly to the overall disease morbidity and mortality [1, 19, 20]. Preliminary findings strongly suggest that IH-AKI, particularly when severe, occur among patients with COVID-19 who have respiratory failure [2]. One explanation for this could be the cytokine storm and heightened inflammatory milieu that occur in COVID-19 patients, and may simultaneously be affecting the lungs and kidneys alike. Hirsh *et al*. [2] reported a 35% mortality among 1993 COVID-19 patients that developed AKI, and in patients requiring renal replacement therapy (RRT) a 55% mortality rate was noted. Some of the reported risk factors for development of AKI in COVID-19 patients include older age, diabetes mellitus, cardiovascular disease (CVD), black race, hypertension, need for mechanical ventilation, need for vasopressor medications, male sex [2], elevated baseline serum creatinine, proteinuria, hematuria, 8 and multiple comorbidities [18]. Our study did not evaluate for these risk factors.

As aforementioned, although the pathogenesis of AKI in COVID-19 is unclear several mechanisms have been proposed. SARS-CoV-2 has been implicated in utilizing the angiotensin converting enzyme-2 (ACE-2) receptor for entry into the cells [21, 22]. These receptors are highly expressed in the proximal tubules of the kidney [21]. The direct viropathic cellular injury that occur [23] may result in tissue damage. Other mechanisms including sepsis, renal hypoperfusion resulting in acute tubular necrosis [20], cytokine storm, alveolar damage due to renal medullary hypoxia, cardiorenal syndrome, and rhabdomyolysis have been reported [22, 24–26]. Immune-mediated glomerulonephritis and primary glomerulopathy resulting in collapsing focal segmental glomerulosclerosis was reported by Magoon *et al*. [27]. Hypercoagulable milieu that occur in COVID-19 may cause thrombotic microangiopathy and result in peritubular and glomerular capillary luminal obstruction [25, 28]. AKI may also occur as a consequence or complication from the treatment of COVID-19. Antiviral agents resulting to tubu-lointerstitial disease [29, 30], and biopsy-proven vitamin C-related oxalate nephropathy have been reported [31]. Certain antibiotics/antibacterial agents have been implicated in AKI in COVID-19 patients [32]. Renal biopsy specimen was not obtained in our study population. Therefore, we did not evaluate the pathogenesis of AKI in the patients included in our study.

AKI among hospitalized COVID-19 patients is associated with increased length of stay and in-hospital deaths, and in patients who survive till hospital discharge AKI increases the risk of developing chronic kidney disease or end-stage kidney disease [1]. Our study did not evaluate the impact of AKI on hospital length of stay but we clearly demonstrate its strong association with in-hospital deaths and adverse cardiopulmonary outcomes though no racial differences in outcomes was noted in subset of patients who have AKI.

The explanation for our finding of null racial differences is uncertain. Given their higher prevalence of comorbidities, including chronic kidney disease, heart failure, hypertension and diabetes mellitus, blacks are at a very high risk of developing COVID-19 related complications, including AKI [18, 33, 34]. Since AKI is associated with higher odds of cardiac events and cardiopulmonary outcomes, and black race have been cited as a risk factor for development of AKI in COVID-19 patients one would expect worse outcomes in blacks. Genetic variants in the Apolipoprotein Li (APOL1) gene, found only in individuals with recent African ancestry, was originally known to confer enhanced innate immunity against African trypanosomes [35]. However, these variants (high-risk genotype) has been strongly implicated with causing high rates of kidney disease in African Americans [35], and have been shown to be associated with focal segmental glomerulosclerosis (FSGS) [27, 36]. In addition, collapsing FSGS has been seen with other viral infections, including parvovirus, cytomegalovirus, and HIV infection, and similar histologic finding of FSGS has also been demonstrated in biopsy specimens of black COVID-19 patients with AKI [27, 36].

Although COVID-19 infection may serve as a second hit to the kidneys in black patients with variants of APOL1 gene resulting in COVID-19 related glomerulopathy and consequent worse outcomes, our study suggests otherwise. However, we noted that more blacks (30%) develop AKI during hospitalization for COVID-19 when compared to non-blacks (18%). It is not unreasonable that a second-hit theory may explain this difference in AKI rates. Till date, we are unaware of any study that has reported similar histologic finding of FSGS in non-black COVID-19 patients with AKI. As this body of evidence continue to grow and evolve it is imperative to understand why blacks with COVID-19 have higher rate of AKI but similar adverse cardiopulmonary outcomes when compared to non-blacks in a subset of patients that developed AKI. A study reported an AKI incidence of 49.3% in a predominantly black population [18]. Although the reported incidence is relatively higher when compared to the previously reported incidences [2, 3, 8] in studies that included predominantly non-black racial population, this study did not find any difference in AKI rates based on race or gender. However, they noted that AKI rate was significantly higher in patients older than 60 years of age when compared to younger subjects [18]. The inability to detect a racial difference in AKI rates in their study may be related to small sample size and therefore study may have been underpowered.

Interestingly, patients who developed AKI were more likely to have a cardiac event suggesting a probable role of cardiorenal interaction in the renal dysfunction that occur in COVID-19. This observation is true regardless of race. The observed association is not unexpected since acute renal failure may result to volume overload and cardiac dysfunction, and vice versa since cardiomyopathy may lead to hypotension, renal hypoperfusion and renal congestion resulting to renal dysfunction [26]. It is also plausible that AKI causing fluid retention, depending on the severity, can acutely worsen clinical cardiac dysfunction and culminate in ARDS. Furthermore, ACE2 receptors are also expressed in the heart and kidneys, providing a link between SARS-CoV-2 infection and the cardiovascular and renal system [23]. Available literature suggests that acute tubular injury, either from ischemic injury or toxic tubular injury, is the most common pathologic finding in autopsy or kidney biopsy specimens of patients with COVID-19 and concomitant AKI [25, 37–40]. Although micro-thrombogenesis, direct viropathic effects and probable toxic effects from pharmacotherapy may contribute to the ischemic acute tubular injury we speculate that renal hypoperfusion due to cardiac dysfunction play an important role. Thus, the need for better understanding of the cardiorenal interaction in COVID-19 patients who develop AKI. Black race, in addition to other risk factors, has been reported as an independent risk factor for COVID-19 associated AKI [41]. Severe COVID-19 disease has been associated with higher frequency of AKI, and IH-AKI has been directly linked with higher death rate in hospitalized patients with COVID-19 [42]. Significant geographic variation in severity and rate of IH-AKI have been reported, and approximately half of the patients with IH-AKI did not completely recover to baseline at the time of discharge [43]. Our study did not evaluate for AKI recovery at the time of discharge.

The findings from our study are similar to those previously reported in prior studies suggesting that AKI is associated with adverse outcomes including in-hospital death [2–4, 44]. In addition, our study provides further evidence that these undesirable outcomes are similar, and not worse, in blacks than non-blacks in the subset of patients who developed in-hospital AKI. We did not find any evidence to suggest racial differences in cardiopulmonary outcomes for COVID-19 patients who develop in-hospital AKI. To our knowledge, no previous study has evaluated the racial differences of cardiopulmonary outcomes in COVID-19 patients who develop AKI.

## 5. Strengths and limitations

Albeit the stated limitations, our study is one of the very few that has addressed racial disparities in outcomes of AKI in the setting of COVID-19 infection. To increase the validity of our results, we had prespecified standardized definitions for all variables including covariates. Our study had a sample size of 267 patients from a single medical center. A more robust multicenter study with a larger sample size is needed to support our findings. Our study is retrospective in nature, we cannot make inferences regarding direct causal relationships. Data was solely abstracted from the electronic medical record system. Because our center carters to a largely black population, there is some variation in the sample sizes of black cohort when compared to the non-black cohort. This may underestimate the event rate in the non-black cohort. Although abstraction was done by trained medical personnel and quality control performed by independent physician investigators, we cannot exclude the possibility of human error in data abstraction. We did not include information from few serological datapoints or variables due to heavy missing values. This limits determination of potential pathogenic mechanisms of the observed associations. We did not evaluate cause-specific mortality. The causes of death may include ARDS, CVD deaths, thromboembolic events from hypercoagulable milieu, acute kidney failure, or MODS, and not necessarily due to AKI. Finally, we excluded patients who were treated on outpatient basis or in the emergency room alone. Therefore, our study findings may be more reflective of those who had a more severe form of COVID-19.

## 6. Conclusions

In COVID-19 patients who develop in-hospital AKI, black race does not pose any additional excess risk of adverse in-hospital cardiopulmonary events or mortality. Regardless of race, in-hospital AKI portends higher risk of in-hospital cardiac events, need for ICU stay, and mechanical ventilation. Blacks with IH-AKI are more likely to have ARDS or die from any cause when compared to blacks without IH-AKI. IH-AKI is a common finding among patients hospitalized with COVID-19 regardless of race. However, development of AKI in the setting of COVID-19 is more common in blacks than non-blacks. Given the aforementioned study limitations, our findings should be interpreted with caution. APOL1 testing and kidney biopsy should be considered in black COVID-19 patients who have rapidly progressive kidney disease in the setting of nephrotic-range proteinuria. Future studies including more data on kidney biopsies will further elucidate alternative or additional pathogenetic mechanisms of AKI in blacks with COVID-19. This may inform and guide management; and may provide alternative explanation for any racial disparities in AKI rates among COVID-19 patients. Identified COVID-19 patients who had in-hospital AKI and survived to hospital discharge should have close clinical follow-up to determine prognosis and long-term impact on the kidneys.

## Figures and Tables

**Table 1.** Baseline characteristics of patients hospitalized with COVID-19 (N = 267).

Variable	All patients (n = 267)	In-hospital AKI (n = 75)	Blacks (n = 217)	Non-blacks (n = 50)
Gender, male	163 (61%)	47 (63%)	127 (59%)	36 (72%)
Age, mean (SD)	61.4 (16.7)	65.9 (14.6)	63.3 (16.3)	53.2 (16.2)
Admission vital signs, mean (SD)				
Systolic blood pressure	134.0 (23.3)	130.7 (22.3)	134.2 (24.0)	133.4 (20.5)
Diastolic blood pressure	79.2 (16.0)	76.8 (16.2)	80.0 (16.5)	75.5 (13.2)
Heart rate	89.7 (18.0)	89.5 (17.1)	90.0 (18.0)	88.5 (18.0)
Respiratory rate	21.6 (9.2)	21.6 (5.5)	21.7 (7.4)	21.3 (14.9)
Pulse oximetry	96.0 (4.9)	94.4 (7.6)	95.9 (5.2)	96.4 (3.5)
Medical/Social history				
Hypertension	192 (72%)	63 (84%)	171 (79%)	21 (42)
Type 2 diabetes	106 (40%)	38 (51%)	91 (42%)	15 (30%)
Dyslipidemia	92 (34%)	33 (44%)	81 (37%)	11 (22%)
CAD	29 (11%)	13 (17%)	27 (12%)	2 (4%)
Atrial fibrillation	25 (9%)	14 (19%)	23 (11%)	2 (4%)
CHF	43 (16%)	22 (29%)	39 (18%)	4 (8%)
VHD	12 (4%)	7 (9%)	12 (6%)	0 (0%)
Prior CVA	46 (17%)	18 (24%)	44 (20%)	2 (4%)
COPD	26 (10%)	8 (11%)	26 (12%)	0 (0%)
Asthma	18 (7%)	4 (5%)	16 (7%)	2 (4%)
Obesity	107 (40%)	36 (48%)	88 (41%)	19 (38%)
HIV	16 (6%)	3 (4%)	12 (6%)	4 (8%)
Chronic liver disease	15 (6%)	7 (9%)	10 (5%)	5 (10%)
Chronic kidney disease	53 (20%)	22 (29%)	44 (20%)	9 (18%)
History of DVT/PE	34 (13%)	10 (13%)	31 (14%)	3 (6%)
Alcohol use	62 (23%)	11 (15%)	48 (22%)	14 (28%)
Tobacco use	76 (28%)	23 (31%)	66 (30%)	10 (20%)
Medications				
Aspirin	79 (30%)	27 (36%)	75 (35%)	4 (8%)
Statin	114 (43%)	41 (55%)	100 (46%)	14 (28%)
Beta-blocker	86 (32%)	36 (48%)	78 (36%)	8 (16%)
ACE-inhibitor	41 (15%)	13 (17%)	36 (17%)	5 (10%)
Angiotensin receptor blocker	27 (10%)	11 (15%)	25 (12%)	2 (4%)
Calcium channel blocker	87 (33%)	29 (39%)	79 (36%)	8 (16%)
P2Y12 platelet inhibitor	16 (6%)	4 (5%)	16 (7%)	0 (0%)

**Table 2.** In-hospital treatment.

Therapy	All (n = 267)	In-hospital AKI (n = 75)	Blacks (n = 217)	Non-blacks (n = 50)
Oxygen, NC	154 (58%)	54 (72%)	127 (59%)	27 (54%)
Non-Invasive ventilation	91 (34%)	41 (55%)	75 (35%)	16 (32%)
Mechanical ventilation	50 (19%)	32 (43%)	41 (19%)	9 (18%)
Corticosteroid	46 (17%)	12 (16%)	37 (17%)	9 (18%)
Hydroxychloroquine	43 (16%)	17 (23%)	36 (17%)	7 (14%)
Azithromycin	71 (27%)	28 (37%)	63 (29%)	8 (16%)
Remdesivir	54 (20%)	16 (21%)	40 (18%)	14 (28%)
Anticoagulation (heparin)	193 (72%)	57 (76%)	160 (74%)	33 (66%)

**Table 3.** Adjusted odds ratio for racial outcomes of in-hospital AKI (N = 75).

Outcome	Blacks (N = 66)	Non-blacks (N = 9)	*OR (95% CI), *p*-value
	n (%)	n (%)	
Primary outcome	42 (64)	7 (78)	0.30 (0.04–1.86), 0.18
ARDS	42 (64)	6 (67)	0.77 (0.16–3.65), 0.74
ICU Stay	33 (50)	5 (56)	0.80 (0.20–3.25), 0.75
Mechanical ventilation	27 (41)	5 (56)	0.51 (0.12–2.10), 0.35
Myocardial injury	36 (55)	5 (56)	0.66 (0.14–3.12), 0.60
In-hospital death	20 (30)	2 (22)	1.40 (0.26–7.50), 0.70

* adjusted OR for race (black vs non-black).

**Table 4.** Adjusted odds ratio for outcomes of in-hospital AKI for various cohorts.

Outcome	All patients (n = 267)	Blacks (n = 222)	Non-blacks (n = 50)
	OR (95% CI), *p*-value	OR (95% CI), *p*-value	OR (95% CI), *p*-value
Primary outcome	2.9 (1.6–5.3), 0.0004	2.7 (1.3–5.5), 0.006	8.6 (1.3–54.8), 0.02
ARDS	4.5 (2.5–8.1), <0.0001	6.4 (3.1–13.1), <0.0001	4.3 (0.9–20.3), 0.07
ICU stay	4.9 (2.7–8.9), <0.0001	6.9 (3.3–14.5), <0.0001	6.1 (1.2–30.7), 0.03
Mechanical ventilation	8.1 (4.0–16.2), <0.0001	11.3 (4.5–28.3), <0.0001	11.2 (2.1–59.9), 0.005
Myocardial injury	2.3 (1.3–4.1), 0.005	2.2 (1.1–4.3), 0.025	4.3 (0.8–22.6), 0.08
In-hospital death	5.7 (2.7–12.1), <0.0001	8.9 (3.3–23.9), <0.0001	11.4 (0.9–143.6), 0.06

**Table 5.** Table of AKI and AKI requiring renal replacement therapy.

AKI requiring renal	AKI		
Replacement Therapy	Yes	No	Total
Yes	14 (18.7%)	-	14
No	61 (81.3%)	192 (100.0%)	253
	75	192	267

**Table 6.** In-hospital mortality among patients with AKI requiring renal replacement therapy.

Race	In-hospital mortality		
	Yes	No	Total
Black	7	4	11
Non-black	1	2	3
	8	6	14

## Abbreviations

CAD: coronary artery disease
CHF: congestive heart failure
VHD: valvular heart disease
CVA: cerebrovascular accident
COPD: chronic obstructive pulmonary disease
HIV: human immunodeficiency virus
DVT: deep venous thrombosis
PE: pulmonary embolism
ACE: angiotensin converting enzyme
COVID-19: Corona Virus Disease-2019
pCVD: Pre-existing cardiovascular disease
AKI: Acute kidney injury
SARS-CoV-2: Severe acute respiratory syndrome coronavirus 2
ARDS: Acute respiratory distress syndrome
IRB: Institutional review board
BMI: Body-mass index
RT-PCR: Reverse-transcriptase-polymerase-chain-reaction
AHF: Acute heart failure
KDIGO: Kidney Disease Improving Global Outcomes
CFR: Case fatality rate
MODS: Multiple organ dysfunction syndrome
ICU: Intensive care unit
SD: Standard deviation
